# Case Report: Clinical and Immunological Features of a Chinese Cohort With *Mycoplasma*-Induced Rash and Mucositis

**DOI:** 10.3389/fped.2020.00402

**Published:** 2020-07-22

**Authors:** Lipin Liu, Ying Wang, Jinqiao Sun, Wenjie Wang, Jia Hou, Xiaochuan Wang

**Affiliations:** ^1^Department of Allergy and Clinical Immunology, Children's Hospital of Fudan University, Shanghai, China; ^2^Department of Pediatrics, Shanghai Songjiang District Central Hospital, Shanghai, China

**Keywords:** *Mycoplasma pneumoniae*, rash, mucositis, lymphopenia, atypical Stevens-Johnson syndrome

## Abstract

Dermatological disorders are the most common extrapulmonary complications of *Mycoplasma pneumoniae*, of which Mycoplasma-induced rash and mucositis (MIRM) has recently been proposed to be a separate diagnostic entity. MIRM could easily be misdiagnosed as atypical Stevens-Johnson syndrome by clinicians due to the unawareness of this rare disease. We retrospectively reviewed the inpatient database from Jan. 2016 to Dec. 2019 of the Children's Hospital of Fudan University. In total, five patients (mean age 5.5 years, three male) matched the diagnostic criteria of MIRM. All patients had scattered lesions and more than two sites of mucosal involvement. The serum IgA level of three patients was higher than normal. Two patients had a significant decrease in peripheral blood CD3+ T and CD4+ T cells that improved with recovery. The percentage of TCRαβ+ CD4–CD8–T cells of Patient five was higher than normal. All patients received treatments with antibiotics and corticosteroids, 3 patients received intravenous immunoglobulin. Among five patients, three patients complained of dyspigmentation, and two patients had an uneventful recovery. MIRM is a separate entity with predominant mucosal involvement and excellent prognosis that more often affects younger patients. Excessive inflammatory reactions may lead to immune disorders, including lymphopenia and a redistribution of CD4+ T cells. We recommend that pneumonia accompanied by mucocutaneous eruptions, especially in young patients, should raise clinical suspicion of MIRM.

## Introduction

*Mycoplasma pneumoniae* is a common cause of respiratory tract infections (1). Although the majority of infections are mild, 25% of patients experience a wide variety of extrapulmonary complications (2). Dermatological disorders, including erythematous, maculopapular and vesicular rashes, are the most common extrapulmonary complications, occurring in up to 25% of patients (1).

Stevens-Johnson syndrome (SJS) and erythema multiforme (EM) have frequently been reported in association with *M. pneumoniae* infection (3, 4). Both of them are thought to be a spectrum of epidermolytic dermopathies (5). Atypical SJS has been described as prominent mucositis and scarce or absent cutaneous involvement (6–10). In 2015, Canavan et al. (11) prompted an additional *Mycoplasma*-induced mucocutaneous eruption entity, presenting as predominant mucositis, with sparse or nonexistent cutaneous involvement, and named it *Mycoplasma*-induced rash and mucositis (MIRM).

The classic MIRM diagnostic criteria are as follows (11): 1. skin detachment <10% of the body surface area (BSA); 2. involvement of at least two mucosal sites; 3. few vesiculobullous lesions or scattered atypical targets; and 4. evidence of *M. pneumoniae* infection. Regarding the last criterion, the evidence of *M. pneumoniae* infection must be supported by the clinical findings of atypical pneumonia, such as fever, cough and positive auscultatory findings, and by laboratory findings, including elevated *M. pneumoniae* IgM antibodies, positive cultures or polymerase chain reaction for *M. pneumoniae* from the oropharynx or bullae, and/or serial cold agglutinins. However, the pathogenesis of MIRM and the reasons of different dermatological manifestations in MP infection are unknown.

MIRM may be formerly have been misdiagnosed as atypical SJS. To date, no MIRM cases have been reported in China. Here, we reported five Chinese cases that matched the MIRM diagnostic criteria and summarized the clinical and immunological characteristics to enhance the awareness of this relatively rare disease.

## Cases Presentation

We retrospectively reviewed the inpatient electronic database from Jan. 2016 to Dec. 2019 in the Children's Hospital of Fudan University. As the entity of “*Mycoplasma* induced rash and mucositis” was not included in the code of the International Classification of Diseases (ICD 10), we searched the database using the terms “Stevens-Johnson syndrome” or “Erythema multiforme” or “Erythema multiforme exudativum” or “Toxic epidermal necrolysis” and overviewed the clinical information of all the above cases. In total, 194 cases were identified and retrospectively analyzed, among which five patients satisfied the MIRM diagnostic criteria proposed by Canavan et al. (11). Of these five patients, three patients were previously diagnosed with “Erythema multiforme,” and another two patients were diagnosed with “Erythema multiforme exudativum.”

### Clinical Presentation

All five patients were previously healthy. None of them had a history of allergic drug exposure or infection prior to the lesion. The mean age was 5.5 years (range: 2 years, 5 months to 7 years), and three patients (60%) were male.

All of the MIRM groups had universal prodromal symptoms preceding the eruption by an average of 2 days (range: 1–4 days), including fever, cough or sore throat. Fever was the main complaint, with a maximum axillary temperature of 39–40°C. Two patients had no obvious respiratory symptoms, while the other three patients had cough during the disease process, and the chest radiograph of two patients suggested bronchial pneumonia (Table 1).

**Table 1:** Demographic characteristics and clinical presentations of *Mycoplasma*-induced rash and mucositis.

	**P1**	**P2**	**P3**	**P4**	**P5**
Age/gender	6 y/F	7 y/M	3 y3 m/F	2 y5 m/M	7 y/M
Previous diagnosis	Erythema multiforme	Erythema multiforme	Erythema multiforme exudativum	Erythema multiforme exudativum	Erythema multiforme
Fever duration	3 days	4 days	5 days	6 days	11 days
Inpatient duration	9 days	4 days	6 days	10 days	11 days
Respiratory symptoms	Slightly cough	No	Cough	No	Cough and sputum
Rash morphology	Macules, maculopapular, vesiculobullous	Macules, targetoid	Macules, maculopapular, vesiculobullous (d = 3 cm)	Maculopapular, vesiculobullous (d = 1 cm)	Maculopapular, vesiculobullous (d = 0.3–1 cm)
Cutaneous distribution	Sparse, <10% BSA	Sparse, <10% BSA	Sparse, <10% BSA	Sparse, <10% BSA	Sparse, <10% BSA
Cutaneous involvement	Face, hands and feet, rare on trunk and limbs	Face, trunk, limbs	Face, trunk, limbs	Face, trunk, limbs	Face, trunk, limbs, penile meatus
Itchiness of rash	/	No itching	Itching	Itching	Itching
Mucosal involvement	Oral, ocular, genital, anus	Oral, ocular	Oral, ocular, genital	Oral, ocular, genital, anal	Oral, ocular, genital
Treatment	Azithromycin, methylprednisolone (22 days), IVIG (2 g/kg), mucosal care	Azithromycin, methylprednisolone (14 days), ceftriaxone	Azithromycin, methylprednisolone (15days)	Azithromycin, methylprednisolone (15 days), IVIG (2 g/kg), acyclovir, cephalosporin, mucosal care;	Azithromycin; methylprednisolone (14 days), IVIG (1 g/kg), acyclovir, cefuroxime, cefoxitin, mucosal care
WBC, × 10^9^/L	7.0	18.5	10.0	6.5	7.6
Neutrophils, %	61.6	90.3	62.1	44.8	82
Lymphocytes, %	24.5	8.7	32.2	46.8	10.1
CRP, mg/L	60	81	36	39	52
ESR, mm/h	20	69	36	54	61
Serum MP-IgM	1:1,280	1:640	>1:1,280	>1:1,280	at admission: N, 1 week later: >1:1,280
Sputum MP-DNA, copies/ml	/	/	/	N	7.48E+06
Clinical outcomes		Full recovery			Full recovery
Dermal:	Dyspigmentation		Dyspigmentation	Dyspigmentation	
Ocular:	Full recovery		Full recovery	Recurrent allergic conjunctivitis	
Urogenital:	Full recovery		Full recovery	Genital synechiae (recovery after surgery)	
Follow-up	29 months	7 months	36 months	32 months	7 months

*M, male; F, female; IVIG, intravenous immunoglobulins; d, diameter; CRP, C-reactive protein; ESR, erythrocyte sedimentation rate; N, negative; /, Not available*.

### Cutaneous Morphology

**Figure 1:**
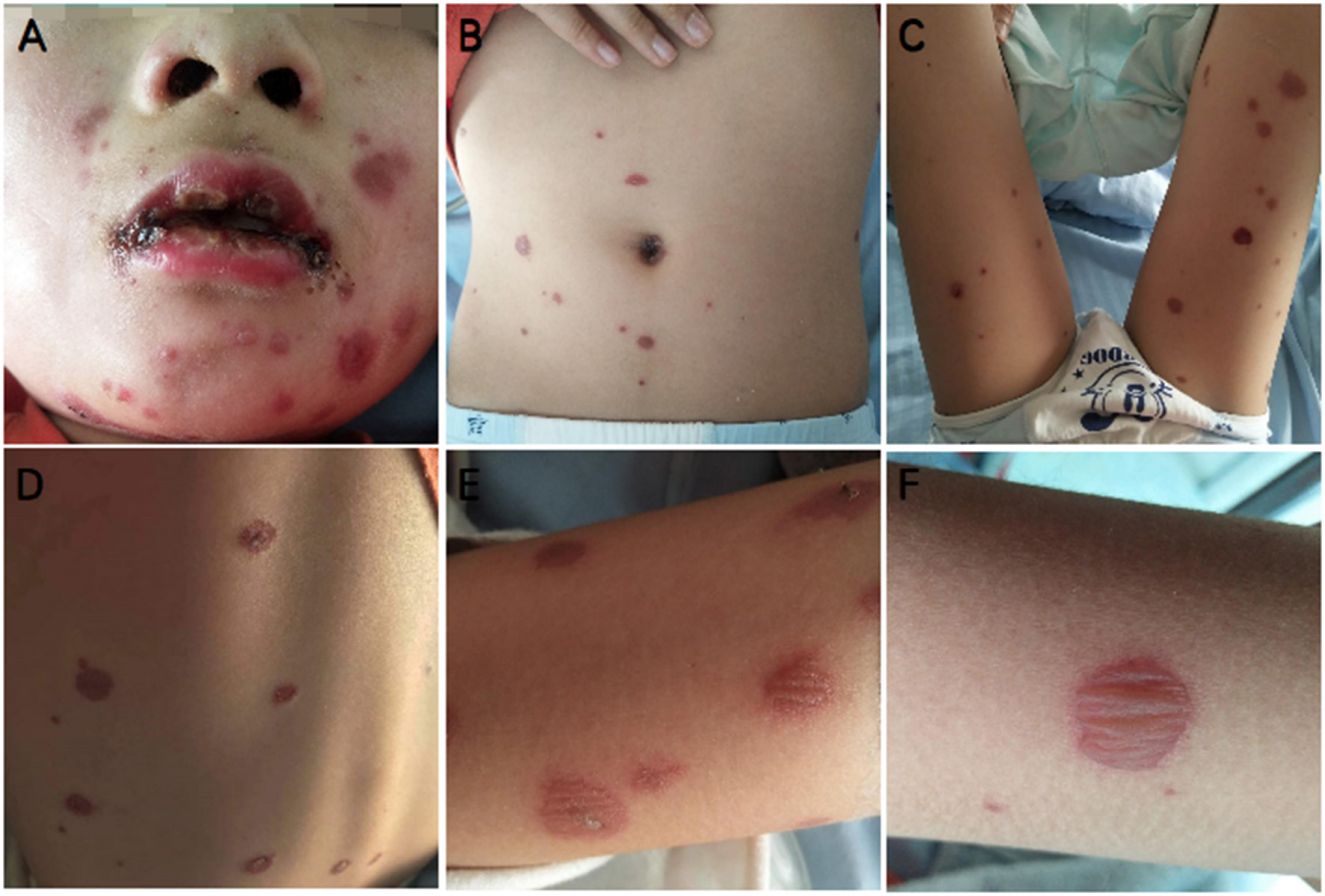
Typical mucocutaneous findings in P5 with *Mycoplasma*-induced rash and mucositis (MIRM). Severe oral mucositis partially covered by hemorrhagic crusts, some atypical targets with central bulla on the face **(A)**, sparse vesiculobullous eruption, and scattered rash on the trunk and limbs **(A–F)**.

All five patients presented with polymorphic lesions (Figure 1). The extent of cutaneous involvement was <10% of the body surface area in all patients. Vesiculobullous and maculopapular rash were the most common morphology (80%), followed by macules (60%) and targetoid lesions (20%). The rash was scattered throughout the body, with some fused into slices, and the vesiculobullous size differed from 0.3 to 3 cm in diameter. They had cutaneous involvement in the same stage of evolution on the face, trunk and limbs; one patient's penile meatus was also involved. The rash of three patients was obvious with itchiness (Table 1).

### Mucositis Morphology

Severe mucositis was present in all five patients, with ≥2 sites of mucosal involvement. The most common mucosal involvement areas were oral and ocular sites (100%), followed by genital (80%) and anal (40%) sites. The clinical observation revealed painful erosions on the lips and oral mucosa that were partially covered by hemorrhagic crusts (Figure 1) as well as bilateral conjunctival hyperemia and genital and perianal mucosal erosion with painful urination or defecation (Table 1).

### Laboratory Findings

Routine blood examinations showed normal or elevated levels of white blood cells (6.5–18.5 × 10^9^/L) with neutrophilia (44.8–90.3%), elevated C-reactive protein levels (29–81 mg/L), and elevated erythrocyte sedimentation rates (20–66 mm/L). Acute herpes simplex virus (HSV) and Epstein-Barr virus infection was excluded in all patients. P5 exhibited a seroconversion of specific *M. pneumoniae* IgM titer (from negative results on admission to a specific *M. pneumoniae* IgM titer ≥1:1,280 at the 2nd examination). The result of sputum pathogen examination showed high *M. pneumoniae* DNA copies of 7.48E+06 copies/ml (normal value <2,500 copies/ml). The remaining four cases had the serological examination at presentation, with a specific *M.pneumoniae* IgM titer ≥1:1,280 in three cases and 1:640 in one case. All patients were excluded other pathogens infection and then diagnosed with *M. pneumoniae* infection (Table 1).

### Immune Function Evaluation

Immune function evaluation was conducted in three patients. The serum IgA level of the three patients was higher than normal, and gradually returned to the normal range after 7 months, while IgM, IgG and IgE were in the normal range. Two patients had a significant decrease in peripheral blood CD3+ and CD4+ T lymphocytes (Table 2). We performed multiple follow-ups of P5 and found that CD3+ T cells significantly increased in 7 months after recovery, and the number of CD4+ T cells, CD8+ T cells and B cells gradually increased (Table 2).

**Table 2:** The immune function of Mycoplasma-induced rash and mucositis.

**Classification**	**P1**	**P2**	**P5**			**References**
			**Level after 2 months**	**Level after 4 months**	**Level after 7 months**	
Subpopulation of lymphocytes, %; (absolute numbers, cells/μL)						
CD3+	61.89 (988.4)	76.97 (1,404)	67.04 (937.80)	65.65 (926.6)	68.79 (1,247)	60.05–74.08 (1,424–2,664)
CD4+	33.89 (541.29)	52.44 (956.7)	38.71 (541.48)	38.68 (546.69)	40.95 (742.36)	26.17–40.76 (686–1,358)
CD8+	26.7 (426.42)	23.27 (424.59)	18.90 (264.32)	17.87 (252.56)	19.17 (347.47)	19.68–34.06 (518–1,125)
CD16+CD56+	6 (95.82)	5.41 (98.77)	16.06 (224.00)	15.60 (220.46)	13.36 (242.36)	9.00–22.24 (258–727)
CD19+	30.56 (488.06)	16.52 (301.41)	13.90 (194.46)	15.60 (220.46)	15.66 (283.82)	10.21–20.12 (280–623)
CD4+/CD8+	1.27	2.25	2.05	2.16	2.14	0.87–1.94
IgG, g/L	8.02	8	6.3	5.2	5.9	5.52–11.4
IgA, g/L	1.87	1.77	0.78	0.71	0.73	0.06–0.74
IgM, g/L	1.8	1.42	0.82	0.68	0.68	0.6–2.12
IgE, KU/L	10.9	63.43	23.57	25.26	63.87	<100

**Figure 2:**
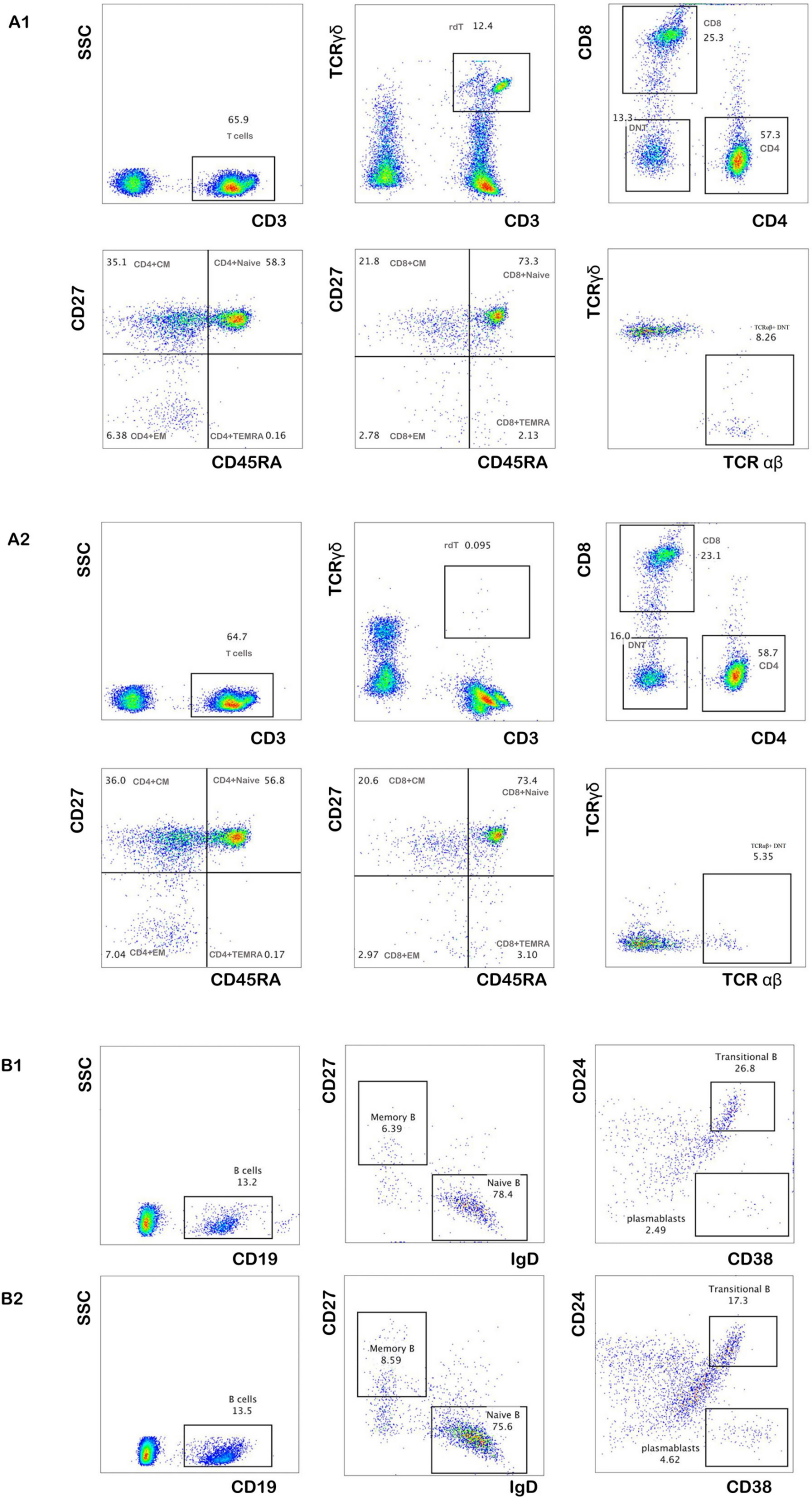
The percentage of peripheral blood lymphocyte subsets of P5. The distribution of the percentage of total T cells and their subsets after 2 and 4 months of follow-up **(A1,A2)**. The distribution of the percentage of total B cells and their subsets after 2 and 4 months of follow-up **(B1,B2)**.

To further explore the immunological features, we performed immunophenotyping of peripheral blood lymphocyte subsets of P5 after 2 and 4 months (Figure 2). The distribution of the percentage of TCRαβ+ CD4–CD8–double-negative (DN) T cells in both experiments was higher than the normal range and gradually decreased with time (Table 3). The distribution of the percentage of transitional B lymphocyte subsets was higher than the normal range, while the remaining B lymphocyte subsets were in the normal range in both experiments (Figure 2, for flow cytometry gating strategy, see Figure S1 in Supplementary File).

**Table 3:** Distribution of the percentage of total T and B cells and their subsets in the peripheral blood of P5 (%).

**Lymphocyte subset**	**Level after 2 months**	**Level after 4 months**	**Reference values, % (12)**
**T cells**			
CD4+Th cells	57.3	58.7	(26.17–40.76)
Naive Th cells	58.3	56.8	(45.56–75.28)
Central memory Th cells	35.1	36	(22.06–46.46)
Effector memory Th cells	6.38	7.04	(2.08–8.78)
Terminal effector memory Th cells	0.16	0.17	(0.00–1.06)
CD8+Cytotoxic T cells	25.3	23.1	(19.68–34.06)
Naive cytotoxic T cells	73.3	73.4	(41.58–77.90)
Central memory cytotoxic T cells	21.8	20.6	(12.08–30.54)
Effector memory cytotoxic T cells	2.78	2.97	(1.58–13.18)
Terminal effector memory cytotoxic T cells	2.13	3.1	(1.70–24.62)
TCRαβ+ double-negative T cells	8.26	5.35	(0.18–2.81)
γ∂ T cells	12.4	0.095	(6.92–19.84)
**B cells**			
Naive B cells	78.4	75.6	(48.36–75.84)
Memory B cells	6.39	8.59	(7.76–19.90)
Transitional B cells	26.8	17.3	(2.58–12.30)
Plasmablasts	2.49	4.62	(0.90–7.36)

### Treatment

All patients received treatments with azithromycin (10 mg/kg IV once daily). Two patients underwent intravenous acyclovir treatment for 5–7 days. Three patients received cephalosporin treatment for 3–10 days. Three patients (60%) with more severe clinical manifestations received IVIG therapy at a dosage of 1–2 g/kg on the second to fifth day of the disease course. These three patients also received local mucosal care. All patients (100%) were treated with corticosteroids. The initial dose of corticosteroids varied between 1.5 and 4 mg/kg depending on the severity of the disease. As clinical symptoms improved, the dose of corticosteroids was gradually reduced, and the total course of corticosteroid treatment was 13–22 days.

After IVIG combined with glucocorticoid therapy, the body temperature of two patients returned to normal within 1–2 days, while the body temperature of P5 returned to normal on the 5th day. Two patients received corticosteroid treatment alone, and the temperature gradually decreased to normal after 2–3 days. All patients had rapid oral, ocular and genital mucositis improvements, and the skin lesions improved.

### Follow-Up and Outcomes

The average duration of hospital stay was 8 days (range 4–11 days). Two patients had a full recovery. The longest follow-up we conducted was 3 years. Dyspigmentation (60%) was the most common skin sequelae. Two patients had double meibomian gland opening embolization and conjunctivitis after 2 weeks. The ocular symptoms of P1 gradually disappeared during follow-up, while P4 had recurrent allergic conjunctivitis. No patients had corneal involvement, and visual outcomes were excellent. One patient (20%) had genital synechiae and underwent circumcision 4 months later.

## Discussion

*Mycoplasma pneumoniae* is the most common infectious agent associated with acute epidermolytic dermopathies (13). The *M. pneumoniae* spectrum of dermatological manifestations varies and includes Raynaud's disease, erythema nodosum, Kawasaki disease, EM and SJS/toxic epidermal necrolysis (TEN) (2, 5). *Mycoplasma*-induced rash and mucositis is rare. Previously, this clinical condition was classified within the epidermolytic dermopathy spectrum as “atypical SJS,” “incomplete SJS,” or “Fuchs syndrome” (5, 10, 11, 14, 15). Recently, a systematic review concluded that this condition was a distinct entity called *Mycoplasma pneumoniae*-induced rash and mucositis (MIRM) (11, 14–17). However, no patient with MIRM has been reported in China until now, indicating that many clinicians had no knowledge of this disease and that such patients could be misdiagnosed.

MIRM is more often characterized by prominent mucositis with a single lesion or a few scattered skin lesions (10, 13, 14, 17, 18), commonly in children and young adolescents (11). The mean age of onset in our cohort was 5.5 years. However, cases of MIRM in young adults have also been reported (16, 19). MIRM is relatively predominant in males (66%) (11), which was similar to our cohort. As reported, all patients had prodromal symptoms by approximately 1 week (11). In our cohort, the eruption appeared approximately on the second day of the disease, earlier than reported in the literature.

Mucosal involvement is critical to the diagnosis, with a mean of 2.5 mucosal sites affected, such as oral mucosal (94%), ocular (82%) and urogenital (63%) sites (11). Other mucosal sites involved include the nares and anus. Mucosal lesions are typically described as ulcerative or hemorrhagic and may be painful (20). Additionally, a cutaneous rash is present in 47% of cases, which are classified as classic MIRM rash. The cutaneous rash of MIRM is sparse in overall distribution and, specifically, is located more in the acral regions (46%) than in the trunk (23%). The cutaneous rash morphology is commonly described as vesiculobullous (77%) and typical target lesions (48%). Less commonly, rashes are described as papules (14%), macules (12%), or morbilliform rashes (9%) (11). The mucocutaneous manifestations of our patients were similar to those of previously reported cases. Interestingly, we found that only the vesiculobullous morphology had obvious itchiness; therefore, some patients were misdiagnosed with varicella in the early stage. Itchiness had not been mentioned in previous literature reports. Therefore, MIRM patients with itchy vesiculobullous lesions should be differentiated from varicella.

Most important is the scarce cutaneous involvement in MIRM. The amount of skin detached is usually <10% BSA, which is critical for clinical distinction. The amount of skin detachment differentiates the extent of SJS/TEN. SJS has <10% skin detachment. Skin detachment of 10–30% indicates SJS/TEN overlap. More than 30% skin detachment indicates TEN (21–23). There are additional features that may help to distinguish MIRM from EM or SJS/TEN. First, the young age characteristic of most patients with MIRM. Second, the etiology of atypical pneumonia in MIRM could be opposed to the HSV associated with EM and the medication etiology of SJS/TEN. It is also notable that there is a better prognosis of MIRM even with support treatment alone (11, 21–25). All these clinical features may help physicians distinguish the clinical spectrum of EM, SJS/TEN and MIRM, where skin lesions are different and milder, explaining its previous and obsolete name of incomplete SJS (11, 26).

Children with *M. pneumoniae* infection may have extrapulmonary manifestations alone. In our study, two patients had no obvious respiratory symptoms. Therefore, serology can help indicating a recent infection with *M. pneumonia* (1). All patients had an *M. pneumoniae*-specific IgM titer higher than normal. We should pay attention to identifying *M. pneumoniae* infection, even if the patient has no respiratory symptoms.

*M. pneumoniae* is a mucosal pathogen; interestingly, several studies demonstrated that IgA antibodies are produced early, peak quickly, and decline earlier than IgM or IgG antibodies (1, 18, 27, 28).

Research on specific T cell-mediated immunity is also involved in the host reaction to *M. pneumoniae* infection, and excessive inflammatory reactions may lead to immune disorders (29–31). By now, it is clear that once *M. pneumoniae* reaches the lower respiratory tract, it can trigger and amplify the production of chemokines promoting lymphocyte and neutrophil tracking and inflammation in the lung. High percentages of neutrophils and lymphocytes are present in alveolar fluid. CD4+ T lymphocytes, B lymphocytes, and plasma cellsinfiltrate the lung (1, 32, 33). Wang et al. (34) reported a case of Stevens-Johnson syndrome associated with *M. pneumoniae* infection, in which lymphopenia, with a significant decrease in CD4+ T cells in the blood and predominant CD4+ T cells in the skin vesicular fluid, was found, and improvement in lymphopenia was associated with disease recovery. In our cohort, we found that two patients with more severe mucocutaneous manifestations had a significant decrease in peripheral blood CD3+ and CD4+ T lymphocytes. Of note, we performed multiple follow-ups of P5 and found that the lymphocyte subsets gradually increased after 7 months, which is similar to the report of Wang et al. However, we did not perform a mucocutaneous biopsy, and the number of lymphocytes in the mucosal lesions is unclear. Therefore, we speculate that patients with MIRM may also have lymphopenia and a redistribution of CD4+ T cells. The mechanism of CD4+ T cell redistribution is not clear.

DNTs are mature post-thymic T cells that express the CD3+TCRαβ+ receptor but lack CD4+CD8+ (35); in healthy humans, approximately 1–5% of all peripheral T cells are of the TCR αβ+DNT phenotype (36, 37). Several studies have demonstrated that various T cell subsets possess immunoregulatory properties (38, 39), and the TCR αβ+ DNT cells was found have the ability to inhibit immune responses (40). Voelkl et al. revealed that human DNT cells exert strong immunosuppressive effects on both CD4+ and CD8+ T cells (40). We performed multiple follow-ups of P5 and found that the distribution of the percentage of TCR αβ+ DNT cells in P5 was higher than normal and gradually decreased with time, and the number of CD4+ T cells, CD8+ T cells gradually increased with disease recovery. Regarding the cause of CD4+ T cell reduction, we speculate that this may be caused by the inhibition of TCR αβ+ DNT cells, and CD4+ T cell redistribution may also play an important role. The distribution of the percentage of transitional B lymphocyte subsets was higher than the normal range, while the remaining B lymphocyte subsets were normal. There were currently no reports on the immunological characteristics of MIRM disease. A limitation is the small number of cases because MIRM remains rare, further investigation is required to reveal the immunological mechanism.

The pathological mechanism of MIRM is currently unclear. MIRM is currently believed to be caused by polyclonal B-cell proliferation, and antibody production may theoretically result in skin damage stemming from immune complex and complement deposition (14, 41, 42). And there was a protein-homeostasis-system hypothesis proposed, it is possible that inflammation-inducing substances in *M. pneumoniae* infection are produced when pathogens are replicated within host cells, including toxins and pathogen-associated molecular patterns (PAMPs), and/or those originated from injured infected-host cells including damage-associated molecular patterns (DAMPs), pathogenic proteins, and pathogenic peptides. When these substances spread systemically and locally and bind to target organ cells, such as pneumonia and other extrapulmonary manifestations were induced, due to the activation of corresponding immune cells and immune proteins, and the substances produced from injured host cells induce further inflammation (43). Importantly, the etiological substances are different from those of EM or SJS/TEN, which are mediated by delayed hypersensitivity reactions and Fas ligand-mediated toxicity (14), leading to the differentiation of MIRM from other cutaneous diseases. Molecular mimicry between *Mycoplasma* P1-adhesion molecules and a keratinocyte antigen has also been speculated (4, 41). The fact that *M. pneumoniae* was isolated from the skin blister fluid on at least two independent occasions could not be ignored, which suggests the possibility of a direct type mechanism (3, 44, 45), and the skin inflammatory bullous might induced by cytokines (2). But in our cohort, the PCR for *M. pneumoniae* from the skin blister fluid of P5 was negative. The pathogenic mechanism and how *M. pneumoniae* transfer from the respiratory tract to the skin need more study.

Currently, there are no guidelines for MIRM treatment. However, most patients are treated with antibiotics, systemic corticosteroids, intravenous immunoglobulin (IVIG), supportive care or a combination of the above (11). Although antibiotic treatment directed at *M. pneumoniae* eliminates the causative agent and limits the duration and severity of the pulmonary disease, the paucity of data does not indicate whether the incidence or severity of the mucocutaneous eruption is reduced (16). Additionally, the role of immunomodulatory therapies is not clear, and there is clinical evidence that IVIG may be helpful for MIRM patients with severe mucositis (11, 14, 17, 20, 26). In our report, three patients with severe mucositis had been treated with IVIG, and only one patient had genital synechiae and underwent circumcision. Future studies would be necessary to investigate the clinical efficacy of immunosuppressants of the aberrant host immune response in cases of severe extrapulmonary manifestations.

## Conclusion

MIRM may a separate clinical entity due to the young age of the patients, the predominant mucosal involvement and the excellent prognosis. Pneumonia accompanied by mucocutaneous eruptions, especially in young patients, should raise clinical suspicion of MIRM. Immunomodulatory therapies may be helpful for MIRM patients with severe mucositis

## Data Availability Statement

The datasets generated for this study are available on request to the corresponding author.

## Ethics Statement

The studies involving human participants were reviewed and approved by Ethics Review Committee of Children's Hospital of Fudan University. Written informed consent to participate in this study was provided by the participants' legal guardian/next of kin. Written informed consent was obtained from the minor(s)' legal guardian/next of kin for the publication of any potentially identifiable images or data included in this article.

## Author Contributions

LL and JH contributed to conceptualize and design the study, conduct the investigation and formal analysis, draft the initial manuscript, and review and revise the manuscript. XW contributed to conceptualize and design the study, supervise the methodology, and critically review and revise the manuscript. WW contributed to the collection the clinical data, conduct investigation, carry out the initial analyses, and review and revise the manuscript. YW and JS contributed to design the study and methodology, supervise clinical data collection, and critically review and revise the manuscript. All authors contributed to the article and approved the submitted version.

## Conflict of Interest

The authors declare that the research was conducted in the absence of any commercial or financial relationships that could be construed as a potential conflict of interest.

## Supplementary Material

Figure S1 Flow cytometry gating strategy for T-cell and B-cell subsets.
