# Supplementary figures and images for: Case Report: Clinical and Immunological Features of a Chinese Cohort With Mycoplasma-Induced Rash and Mucositis
Source: Front Pediatr. 2020 Jul 22;8:402. doi: 10.3389/fped.2020.00402 (PMC7387509; doi:10.3389/fped.2020.00402)

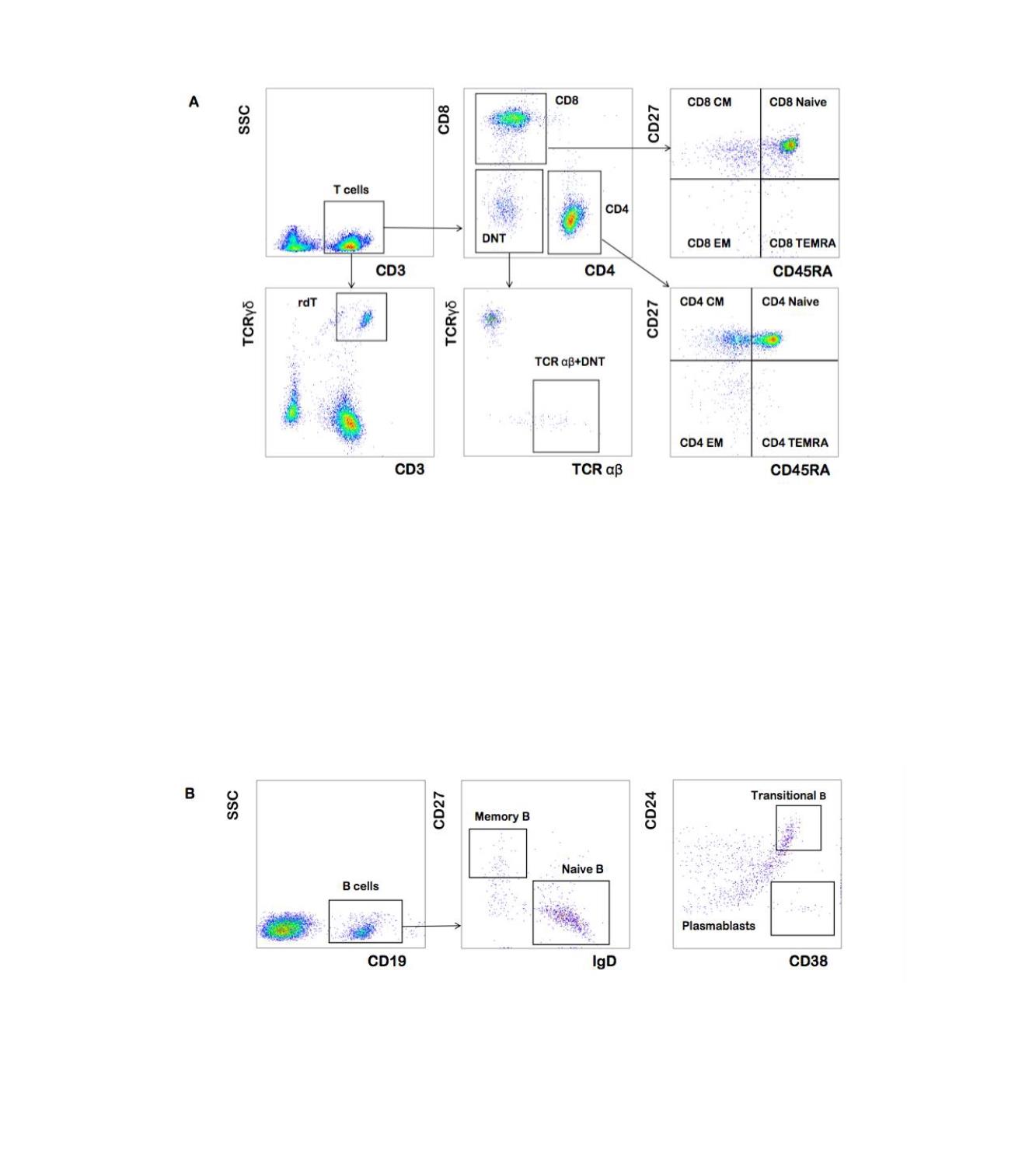

Supplement: Figure S1 — Flow cytometry gating strategy for T-cell and B-cell subsets. [file Image_1.JPEG]
